# Poly[(μ_4_-3-carb­oxy­benzoato-κ^5^ *O* ^1^:*O* ^1^,*O* ^1′^:*O* ^1′^:*O* ^3^)(quinolin-8-olato-κ^2^ *N*,*O*)lead(II)]

**DOI:** 10.1107/S160053681105495X

**Published:** 2012-01-07

**Authors:** Akbar Ghaemi, Zohreh Dadkhah, Seik Weng Ng, Edward R. T. Tiekink

**Affiliations:** a Department of Chemistry, Saveh Branch, Islamic Azad University, Saveh, Iran; b Department of Chemistry, University of Malaya, 50603 Kuala Lumpur, Malaysia; c Chemistry Department, Faculty of, Science, King Abdulaziz University, PO Box 80203 Jeddah, Saudi Arabia

## Abstract

The asymmetric unit of the title complex, [Pb(C_8_H_5_O_4_)(C_9_H_6_NO)]_*n*_, comprises a Pb^II^ cation, a quinolin-8-olate anion and a 3-carb­oxy­benzoate anion. The coordination geometry of the Pb^II^ atom is defined by one N and six O atoms, as well as a stereochemically active lone pair of electrons, and is based on a Ψ-dodeca­hedron. The quinolin-8-olate is chelating and the 3-carb­oxy­benzoate anion forms bonds to four different Pb^II^ atoms. The benzoate end of the 3-carb­oxy­benzoate ligand chelates one Pb^II^ atom and simultaneously bridges two Pb^II^ atoms on either side, forming a chain along the *b* axis. The carboxyl end of the 3-carb­oxy­benzoate connects to a neighbouring chain by employing its carbonyl atom to form a bond to a Pb^II^ atom and the hydroxyl group to form a hydrogen bond to a quinolin-8-olate O atom. Thereby, a layer is formed in the *bc* plane.

## Related literature

For background to Pb^II^ mixed quinolate carboxyl­ate structures, see: Shahverdizadeh *et al.* (2008 ▶).
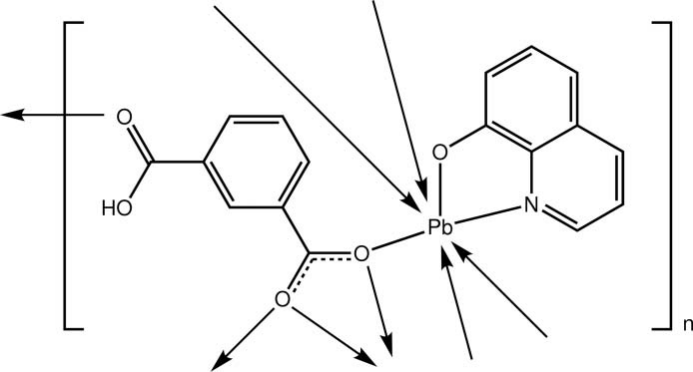


## Experimental

### 

#### Crystal data

[Pb(C_8_H_5_O_4_)(C_9_H_6_NO)]
*M* *_r_* = 516.46
Monoclinic, 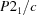

*a* = 9.0746 (2) Å
*b* = 7.0262 (2) Å
*c* = 22.6919 (6) Å
β = 93.185 (3)°
*V* = 1444.60 (6) Å^3^
*Z* = 4
Mo *K*α radiation
μ = 11.71 mm^−1^
*T* = 100 K
0.25 × 0.20 × 0.15 mm

#### Data collection

Agilent SuperNova Dual diffractometer with an Atlas detector
Absorption correction: multi-scan (*CrysAlis PRO*; Agilent, 2010 ▶) *T*_min_ = 0.158, *T*_max_ = 0.273
9690 measured reflections
3325 independent reflections
3035 reflections with *I* > 2σ(*I*)
*R*_int_ = 0.029

#### Refinement

*R*[*F* ^2^ > 2σ(*F* ^2^)] = 0.021
*wR*(*F* ^2^) = 0.049
*S* = 1.01
3325 reflections
221 parameters
1 restraint
H atoms treated by a mixture of independent and constrained refinement
Δρ_max_ = 0.92 e Å^−3^
Δρ_min_ = −1.27 e Å^−3^

### 

Data collection: *CrysAlis PRO* (Agilent, 2010 ▶); cell refinement: *CrysAlis PRO*; data reduction: *CrysAlis PRO*; program(s) used to solve structure: *SHELXS97* (Sheldrick, 2008 ▶); program(s) used to refine structure: *SHELXL97* (Sheldrick, 2008 ▶); molecular graphics: *ORTEP-3* (Farrugia, 1997 ▶) and *DIAMOND* (Brandenburg, 2006 ▶); software used to prepare material for publication: *publCIF* (Westrip, 2010 ▶).

## Supplementary Material

Crystal structure: contains datablock(s) global, I. DOI: 10.1107/S160053681105495X/qm2046sup1.cif

Structure factors: contains datablock(s) I. DOI: 10.1107/S160053681105495X/qm2046Isup2.hkl

Additional supplementary materials:  crystallographic information; 3D view; checkCIF report

## Figures and Tables

**Table 1:** Selected bond lengths (Å)

Pb—O1	2.608 (2)
Pb—O1^i^	2.746 (2)
Pb—O2^ii^	2.578 (2)
Pb—O2^i^	2.809 (2)
Pb—O3^iii^	2.840 (3)
Pb—O5	2.318 (2)
Pb—N1	2.468 (3)

Symmetry codes: (i) 
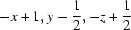
; (ii) 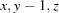; (iii) 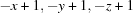.

**Table 2:** Hydrogen-bond geometry (Å, °)

*D*—H⋯*A*	*D*—H	H⋯*A*	*D*⋯*A*	*D*—H⋯*A*
O4—H1⋯O5^iii^	0.84 (1)	1.74 (3)	2.539 (4)	158 (6)

Symmetry code: (iii) 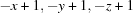.

**Fig. 1.**
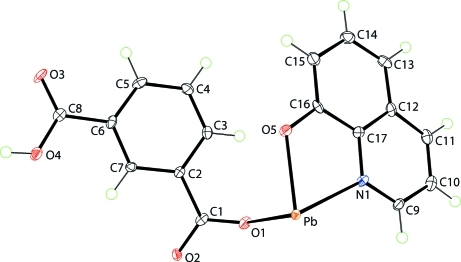
The asymmetric unit of (I) showing the atom-labelling scheme and displacement ellipsoids at the 50% probability level.

**Fig. 2.**
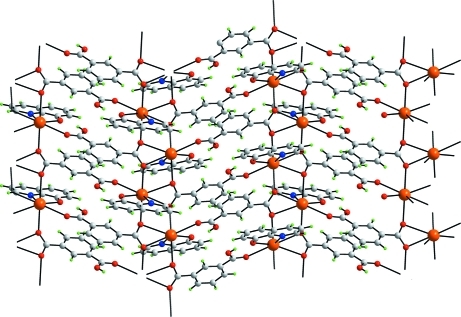
A view of the layer in the bc plane in (I).

**Fig. 3.**
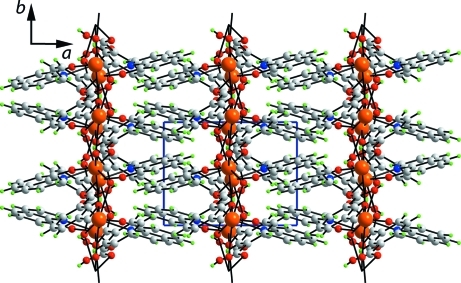
A view in projection down the c axis of the unit-cell contents of (I) highlighting the stacking of layers.

## supplementary crystallographic information

### Comment

Mixed lead(II) complexes of quinolin-8-olate and monofunctional carboxylates have displayed a variety of structural motifs (Shahverdizadeh *et al.*, 2008). In the present report, a 1:1 structure containing quinolin-8-olate and 3-carboxybenzoate is described, (I).

The asymmetric unit of (I) comprises a Pb^II^ cation, a quinolin-8-olate anion and a 3-carboxybenzoate anion, Fig. 1. The coordination geometry of the Pb^II^ atom is defined by a N and six O atoms as well as a stereochemically active lone pair of electrons, and is based on a Ψ-dodecahedron. The quinolin-8-olate anion is chelating, whereas the 3-carboxybenzoate anion is pentadentate, forming bonds to four different Pb^II^ atoms, Table 1. The benzoate group chelates one Pb^II^ atom and each of these O atoms forms a bond to a neighbouring Pb^II^ to form a chain along the *b* axis. Adjacent chains, along the *c* axis, are connected by Pb—*O*(carbonyl) bonds. The hydroxyl group forms a hydrogen bond to the quinolin-8-olate-O atom, Table 2. The result is a layer in the *bc* plane. Layers stack along the *a* axis, Fig. 3, with no specific intermolecular interactions between them.

### Experimental

The title complex was obtained by the following method. A methanol solution (10 ml) of 8-hydroxyquinoline (0.145 g, 1 mmol) was added to an aqueous solution (2 ml) of Pb(NO_3_)_2_ (0.331 g, 1 mmol). The mixture was stirred for 10 min. To this solution, was added a DMF solution (5 ml) of isophthalic acid (0.084 g, 0.5 mmol) slowly at room temperature. This mixture was filtered. After keeping the filtrate in air, crystals were formed at the bottom of the vessel upon slow evaporation of the solvents at room temperature. *M*.pt. 558 K (dec.). Yield: 65%.

### Refinement

Carbon-bound H-atoms were placed in calculated positions [C—H 0.95 Å, *U*_iso_(H) 1.2*U*_eq_(C)] and were included in the refinement in the riding model approximation. The acid H-atom was located in a difference Fourier map, and was refined with a distance restraint of O—H 0.84±0.01 Å; its *U*_iso_ value was refined. The final difference Fourier map had a peak at 0.81 Å from Pb and a hole at 0.90 Å from the same atom.

### Crystal data

**Table:**

[Pb(C_8_H_5_O_4_)(C_9_H_6_NO)]	*F*(000) = 968
*M**_r_* = 516.46	*D*_x_ = 2.375 Mg m^−^^3^
Monoclinic, *P*2_1_/*c*	Mo *K*α radiation, λ = 0.71073 Å
Hall symbol: -P 2ybc	Cell parameters from 6118 reflections
*a* = 9.0746 (2) Å	θ = 2.2–27.5°
*b* = 7.0262 (2) Å	µ = 11.71 mm^−^^1^
*c* = 22.6919 (6) Å	*T* = 100 K
β = 93.185 (3)°	Block, yellow
*V* = 1444.60 (6) Å^3^	0.25 × 0.20 × 0.15 mm
*Z* = 4	

### Data collection

**Table:**

Agilent SuperNova Dual diffractometer with an Atlas detector	3325 independent reflections
Radiation source: SuperNova (Mo) X-ray Source	3035 reflections with *I* > 2σ(*I*)
Mirror	*R*_int_ = 0.029
Detector resolution: 10.4041 pixels mm^-1^	θ_max_ = 27.6°, θ_min_ = 2.8°
ω scan	*h* = −11→11
Absorption correction: multi-scan (CrysAlis PRO; Agilent, 2010)	*k* = −9→8
*T*_min_ = 0.158, *T*_max_ = 0.273	*l* = −29→27
9690 measured reflections	

### Refinement

**Table:**

Refinement on *F*^2^	Primary atom site location: structure-invariant direct methods
Least-squares matrix: full	Secondary atom site location: difference Fourier map
*R*[*F*^2^ > 2σ(*F*^2^)] = 0.021	Hydrogen site location: inferred from neighbouring sites
*wR*(*F*^2^) = 0.049	H atoms treated by a mixture of independent and constrained refinement
*S* = 1.01	*w* = 1/[σ^2^(*F*_o_^2^) + (0.0237*P*)^2^ + 0.5749*P*] where *P* = (*F*_o_^2^ + 2*F*_c_^2^)/3
3325 reflections	(Δ/σ)_max_ = 0.001
221 parameters	Δρ_max_ = 0.92 e Å^−^^3^
1 restraint	Δρ_min_ = −1.27 e Å^−^^3^

### Fractional atomic coordinates and isotropic or equivalent isotropic displacement parameters (Å^2^)

**Table:**

	*x*	*y*	*z*	*U*_iso_*/*U*_eq_	
Pb	0.513121 (14)	0.057458 (17)	0.305356 (5)	0.00777 (5)	
O1	0.5980 (3)	0.4114 (3)	0.30020 (11)	0.0123 (5)	
O2	0.4886 (3)	0.6925 (3)	0.31117 (10)	0.0107 (5)	
O3	0.6052 (3)	0.7915 (4)	0.59124 (11)	0.0160 (6)	
O4	0.4185 (3)	0.8397 (4)	0.52393 (12)	0.0141 (5)	
H1	0.365 (6)	0.890 (8)	0.549 (2)	0.08 (2)*	
O5	0.6896 (3)	0.0346 (3)	0.38285 (11)	0.0111 (5)	
N1	0.7528 (4)	−0.0485 (4)	0.27073 (13)	0.0104 (6)	
C1	0.5716 (4)	0.5570 (5)	0.33079 (16)	0.0099 (7)	
C2	0.6368 (4)	0.5693 (4)	0.39260 (16)	0.0084 (7)	
C3	0.7672 (4)	0.4692 (5)	0.40797 (16)	0.0112 (7)	
H3	0.8141	0.3966	0.3791	0.013*	
C4	0.8270 (4)	0.4767 (5)	0.46522 (17)	0.0136 (8)	
H4	0.9160	0.4104	0.4754	0.016*	
C5	0.7586 (4)	0.5801 (5)	0.50806 (17)	0.0140 (8)	
H5	0.8006	0.5848	0.5473	0.017*	
C6	0.6275 (4)	0.6774 (5)	0.49317 (15)	0.0111 (7)	
C7	0.5672 (4)	0.6744 (5)	0.43551 (15)	0.0100 (7)	
H7	0.4794	0.7432	0.4253	0.012*	
C8	0.5498 (4)	0.7761 (5)	0.54049 (16)	0.0109 (7)	
C9	0.7848 (4)	−0.0880 (5)	0.21594 (16)	0.0116 (8)	
H9	0.7081	−0.0799	0.1858	0.014*	
C10	0.9255 (4)	−0.1409 (5)	0.19995 (17)	0.0149 (8)	
H10	0.9432	−0.1670	0.1599	0.018*	
C11	1.0383 (4)	−0.1548 (5)	0.24300 (16)	0.0140 (8)	
H11	1.1352	−0.1870	0.2327	0.017*	
C12	1.0090 (4)	−0.1206 (5)	0.30261 (16)	0.0117 (8)	
C13	1.1178 (4)	−0.1327 (5)	0.34965 (17)	0.0148 (8)	
H13	1.2155	−0.1707	0.3422	0.018*	
C14	1.0821 (4)	−0.0899 (5)	0.40557 (18)	0.0156 (8)	
H14	1.1558	−0.0983	0.4369	0.019*	
C15	0.9372 (4)	−0.0329 (5)	0.41800 (18)	0.0144 (8)	
H15	0.9155	−0.0037	0.4575	0.017*	
C16	0.8271 (4)	−0.0193 (5)	0.37373 (17)	0.0106 (7)	
C17	0.8635 (4)	−0.0657 (4)	0.31489 (16)	0.0098 (7)	

### Atomic displacement parameters (Å^2^)

**Table:**

	*U*^11^	*U*^22^	*U*^33^	*U*^12^	*U*^13^	*U*^23^
Pb	0.00854 (8)	0.00841 (7)	0.00647 (8)	0.00005 (5)	0.00123 (5)	−0.00047 (5)
O1	0.0151 (14)	0.0122 (12)	0.0097 (13)	−0.0020 (11)	0.0002 (11)	−0.0010 (10)
O2	0.0123 (13)	0.0092 (12)	0.0107 (13)	0.0024 (10)	0.0018 (10)	−0.0005 (10)
O3	0.0171 (14)	0.0249 (14)	0.0061 (13)	−0.0028 (12)	0.0007 (11)	−0.0049 (11)
O4	0.0134 (14)	0.0197 (13)	0.0093 (13)	0.0005 (12)	0.0022 (11)	−0.0020 (11)
O5	0.0098 (13)	0.0145 (13)	0.0089 (13)	−0.0005 (10)	0.0005 (10)	−0.0008 (10)
N1	0.0118 (16)	0.0112 (15)	0.0080 (16)	−0.0034 (12)	−0.0002 (13)	−0.0007 (11)
C1	0.0112 (18)	0.0116 (17)	0.0072 (18)	−0.0050 (14)	0.0035 (14)	0.0023 (13)
C2	0.0123 (18)	0.0057 (15)	0.0074 (17)	−0.0025 (14)	0.0017 (14)	0.0017 (12)
C3	0.0126 (19)	0.0089 (16)	0.0123 (19)	−0.0005 (15)	0.0036 (15)	−0.0008 (14)
C4	0.0088 (18)	0.0169 (18)	0.015 (2)	0.0027 (15)	−0.0003 (15)	0.0001 (15)
C5	0.013 (2)	0.0189 (18)	0.0094 (19)	−0.0046 (16)	−0.0021 (15)	−0.0007 (15)
C6	0.0154 (19)	0.0104 (16)	0.0080 (18)	−0.0011 (15)	0.0045 (14)	0.0004 (14)
C7	0.0110 (18)	0.0084 (16)	0.0103 (18)	0.0000 (14)	−0.0031 (14)	−0.0012 (13)
C8	0.0120 (18)	0.0099 (16)	0.0108 (18)	−0.0054 (15)	0.0015 (14)	−0.0001 (14)
C9	0.0149 (19)	0.0112 (16)	0.0087 (18)	−0.0008 (15)	−0.0008 (15)	−0.0019 (14)
C10	0.020 (2)	0.0126 (18)	0.0129 (19)	−0.0008 (16)	0.0050 (15)	−0.0023 (14)
C11	0.0153 (19)	0.0098 (16)	0.017 (2)	0.0002 (15)	0.0062 (15)	0.0008 (14)
C12	0.0111 (19)	0.0068 (16)	0.017 (2)	−0.0019 (14)	0.0016 (15)	−0.0027 (14)
C13	0.0078 (18)	0.0150 (17)	0.021 (2)	0.0017 (15)	0.0003 (15)	0.0032 (15)
C14	0.012 (2)	0.0176 (18)	0.017 (2)	−0.0007 (16)	−0.0034 (16)	−0.0005 (15)
C15	0.014 (2)	0.0127 (18)	0.017 (2)	0.0015 (15)	0.0002 (16)	−0.0009 (15)
C16	0.0125 (19)	0.0031 (15)	0.016 (2)	0.0012 (14)	0.0022 (15)	0.0027 (13)
C17	0.0106 (18)	0.0071 (16)	0.0116 (19)	−0.0027 (14)	−0.0006 (15)	−0.0003 (13)

### Geometric parameters (Å, °)

**Table:**

Pb—O1	2.608 (2)	C4—C5	1.388 (5)
Pb—O1^i^	2.746 (2)	C4—H4	0.9500
Pb—O2^ii^	2.578 (2)	C5—C6	1.397 (5)
Pb—O2^i^	2.809 (2)	C5—H5	0.9500
Pb—O3^iii^	2.840 (3)	C6—C7	1.390 (5)
Pb—O5	2.318 (2)	C6—C8	1.489 (5)
Pb—N1	2.468 (3)	C7—H7	0.9500
O1—C1	1.267 (4)	C9—C10	1.397 (5)
O1—Pb^iv^	2.746 (2)	C9—H9	0.9500
O2—C1	1.278 (4)	C10—C11	1.379 (5)
O2—Pb^v^	2.578 (2)	C10—H10	0.9500
O3—C8	1.235 (4)	C11—C12	1.414 (5)
O4—C8	1.308 (4)	C11—H11	0.9500
O4—H1	0.840 (10)	C12—C13	1.416 (5)
O5—C16	1.331 (4)	C12—C17	1.418 (5)
N1—C9	1.322 (5)	C13—C14	1.360 (5)
N1—C17	1.385 (4)	C13—H13	0.9500
C1—C2	1.494 (5)	C14—C15	1.418 (6)
C2—C3	1.403 (5)	C14—H14	0.9500
C2—C7	1.400 (5)	C15—C16	1.380 (5)
C3—C4	1.381 (5)	C15—H15	0.9500
C3—H3	0.9500	C16—C17	1.431 (5)
			
O5—Pb—N1	68.69 (10)	C6—C5—H5	120.2
O5—Pb—O2^ii^	87.14 (7)	C7—C6—C5	120.4 (3)
N1—Pb—O2^ii^	78.24 (8)	C7—C6—C8	120.6 (3)
O5—Pb—O1	84.66 (8)	C5—C6—C8	119.0 (3)
N1—Pb—O1	90.33 (9)	C6—C7—C2	119.6 (3)
O2^ii^—Pb—O1	167.78 (8)	C6—C7—H7	120.2
O5—Pb—O1^i^	147.23 (8)	C2—C7—H7	120.2
N1—Pb—O1^i^	84.08 (9)	O3—C8—O4	123.7 (3)
O2^ii^—Pb—O1^i^	69.16 (7)	O3—C8—C6	121.9 (3)
O1—Pb—O1^i^	114.35 (6)	O4—C8—C6	114.4 (3)
O5—Pb—O3^iii^	71.10 (8)	N1—C9—C10	123.5 (3)
N1—Pb—O3^iii^	139.12 (8)	N1—C9—H9	118.2
O2^ii^—Pb—O3^iii^	106.98 (8)	C10—C9—H9	118.2
O1—Pb—O3^iii^	78.86 (8)	C11—C10—C9	119.2 (4)
O1^i^—Pb—O3^iii^	136.32 (8)	C11—C10—H10	120.4
C1—O1—Pb	132.9 (2)	C9—C10—H10	120.4
C1—O1—Pb^iv^	96.0 (2)	C10—C11—C12	119.6 (4)
Pb—O1—Pb^iv^	107.49 (8)	C10—C11—H11	120.2
C1—O2—Pb^v^	135.0 (2)	C12—C11—H11	120.2
C8—O4—H1	120 (4)	C11—C12—C13	123.4 (4)
C16—O5—Pb	121.1 (2)	C11—C12—C17	117.5 (3)
C9—N1—C17	118.3 (3)	C13—C12—C17	119.1 (3)
C9—N1—Pb	127.4 (2)	C14—C13—C12	119.9 (4)
C17—N1—Pb	114.4 (2)	C14—C13—H13	120.1
O1—C1—O2	122.4 (3)	C12—C13—H13	120.1
O1—C1—C2	118.9 (3)	C13—C14—C15	121.3 (4)
O2—C1—C2	118.6 (3)	C13—C14—H14	119.4
C3—C2—C7	119.9 (3)	C15—C14—H14	119.4
C3—C2—C1	119.3 (3)	C16—C15—C14	121.1 (4)
C7—C2—C1	120.9 (3)	C16—C15—H15	119.5
C4—C3—C2	119.8 (4)	C14—C15—H15	119.5
C4—C3—H3	120.1	O5—C16—C15	123.6 (4)
C2—C3—H3	120.1	O5—C16—C17	118.5 (3)
C3—C4—C5	120.7 (4)	C15—C16—C17	117.9 (3)
C3—C4—H4	119.6	N1—C17—C12	121.9 (3)
C5—C4—H4	119.6	N1—C17—C16	117.4 (3)
C4—C5—C6	119.7 (3)	C12—C17—C16	120.7 (3)
C4—C5—H5	120.2		
			
O5—Pb—O1—C1	−77.9 (3)	C3—C4—C5—C6	0.2 (6)
N1—Pb—O1—C1	−146.5 (3)	C4—C5—C6—C7	−1.4 (5)
O2^ii^—Pb—O1—C1	−125.9 (4)	C4—C5—C6—C8	175.7 (3)
O1^i^—Pb—O1—C1	129.9 (3)	C5—C6—C7—C2	1.5 (5)
O3^iii^—Pb—O1—C1	−6.2 (3)	C8—C6—C7—C2	−175.5 (3)
O5—Pb—O1—Pb^iv^	165.82 (11)	C3—C2—C7—C6	−0.5 (5)
N1—Pb—O1—Pb^iv^	97.27 (11)	C1—C2—C7—C6	177.6 (3)
O2^ii^—Pb—O1—Pb^iv^	117.8 (3)	C7—C6—C8—O3	−175.3 (3)
O1^i^—Pb—O1—Pb^iv^	13.62 (6)	C5—C6—C8—O3	7.6 (5)
O3^iii^—Pb—O1—Pb^iv^	−122.43 (10)	C7—C6—C8—O4	6.3 (5)
N1—Pb—O5—C16	0.4 (2)	C5—C6—C8—O4	−170.8 (3)
O2^ii^—Pb—O5—C16	78.9 (2)	C17—N1—C9—C10	−1.9 (5)
O1—Pb—O5—C16	−92.1 (2)	Pb—N1—C9—C10	177.7 (3)
O1^i^—Pb—O5—C16	36.2 (3)	N1—C9—C10—C11	0.4 (5)
O3^iii^—Pb—O5—C16	−172.1 (2)	C9—C10—C11—C12	1.8 (5)
O5—Pb—N1—C9	−179.4 (3)	C10—C11—C12—C13	179.6 (3)
O2^ii^—Pb—N1—C9	89.1 (3)	C10—C11—C12—C17	−2.4 (5)
O1—Pb—N1—C9	−95.3 (3)	C11—C12—C13—C14	177.2 (3)
O1^i^—Pb—N1—C9	19.2 (3)	C17—C12—C13—C14	−0.7 (5)
O3^iii^—Pb—N1—C9	−168.5 (2)	C12—C13—C14—C15	0.0 (6)
O5—Pb—N1—C17	0.1 (2)	C13—C14—C15—C16	0.1 (6)
O2^ii^—Pb—N1—C17	−91.4 (2)	Pb—O5—C16—C15	179.1 (3)
O1—Pb—N1—C17	84.3 (2)	Pb—O5—C16—C17	−0.9 (4)
O1^i^—Pb—N1—C17	−161.3 (2)	C14—C15—C16—O5	−179.5 (3)
O3^iii^—Pb—N1—C17	11.0 (3)	C14—C15—C16—C17	0.4 (5)
Pb—O1—C1—O2	−108.0 (4)	C9—N1—C17—C12	1.1 (5)
Pb^iv^—O1—C1—O2	12.7 (4)	Pb—N1—C17—C12	−178.5 (3)
Pb—O1—C1—C2	70.5 (4)	C9—N1—C17—C16	179.0 (3)
Pb^iv^—O1—C1—C2	−168.8 (3)	Pb—N1—C17—C16	−0.6 (4)
Pb^v^—O2—C1—O1	−129.2 (3)	C11—C12—C17—N1	1.0 (5)
Pb^v^—O2—C1—C2	52.3 (5)	C13—C12—C17—N1	179.0 (3)
O1—C1—C2—C3	25.8 (5)	C11—C12—C17—C16	−176.8 (3)
O2—C1—C2—C3	−155.6 (3)	C13—C12—C17—C16	1.2 (5)
O1—C1—C2—C7	−152.3 (3)	O5—C16—C17—N1	1.0 (5)
O2—C1—C2—C7	26.2 (5)	C15—C16—C17—N1	−179.0 (3)
C7—C2—C3—C4	−0.7 (5)	O5—C16—C17—C12	178.9 (3)
C1—C2—C3—C4	−178.9 (3)	C15—C16—C17—C12	−1.1 (5)
C2—C3—C4—C5	0.9 (6)		

Symmetry codes: (i) −*x*+1, *y*−1/2, −*z*+1/2; (ii) *x*, *y*−1, *z*; (iii) −*x*+1, −*y*+1, −*z*+1; (iv) −*x*+1, *y*+1/2, −*z*+1/2; (v) *x*, *y*+1, *z*.

### Hydrogen-bond geometry (Å, °)

**Table:**

*D*—H···*A*	*D*—H	H···*A*	*D*···*A*	*D*—H···*A*
O4—H1···O5^iii^	0.84 (1)	1.74 (3)	2.539 (4)	158 (6)

Symmetry codes: (iii) −*x*+1, −*y*+1, −*z*+1.

## References

[bb1] Agilent (2010). *CrysAlis PRO* Agilent Technologies, Yarnton, England.

[bb2] Brandenburg, K. (2006). *DIAMOND* Crystal Impact GbR, Bonn, Germany.

[bb3] Farrugia, L. J. (1997). *J. Appl. Cryst.* **30**, 565.

[bb4] Shahverdizadeh, G. H., Soudi, A. A., Morsali, A. & Retailleau, P. (2008). *Inorg. Chim. Acta*, **361**, 1875–1884.

[bb5] Sheldrick, G. M. (2008). *Acta Cryst.* A**64**, 112–122. 10.1107/S0108767307043930 18156677

[bb6] Westrip, S. P. (2010). *J. Appl. Cryst.* **43**, 920–925.

